# Surprising results on phylogenetic tree building methods based on molecular sequences

**DOI:** 10.1186/1471-2105-13-148

**Published:** 2012-06-27

**Authors:** Gaston H Gonnet

**Affiliations:** 1 Department of Computer Science, ETH Zurich, Zurich, Switzerland

**Keywords:** Phylogenetic trees, Tree building methods, Maximum likelihood, Distance measures, Multiple sequence alignments, Substitution matrices, Molecular sequences

## Abstract

**Background:**

We analyze phylogenetic tree building methods from molecular sequences (PTMS). These are methods which base their construction solely on sequences, coding DNA or amino acids.

**Results:**

Our first result is a statistically significant evaluation of 176 PTMSs done by comparing trees derived from 193138 orthologous groups of proteins using a new measure of quality between trees. This new measure, called the Intra measure, is very consistent between different groups of species and strong in the sense that it separates the methods with high confidence.

The second result is the comparison of the trees against trees derived from accepted taxonomies, the Taxon measure. We consider the NCBI taxonomic classification and their derived topologies as the most accepted biological consensus on phylogenies, which are also available in electronic form. The correlation between the two measures is remarkably high, which supports both measures simultaneously.

**Conclusions:**

The big surprise of the evaluation is that the maximum likelihood methods do not score well, minimal evolution distance methods over MSA-induced alignments score consistently better. This comparison also allows us to rank different components of the tree building methods, like MSAs, substitution matrices, ML tree builders, distance methods, etc. It is also clear that there is a difference between Metazoa and the rest, which points out to evolution leaving different molecular traces. We also think that these measures of quality of trees will motivate the design of new PTMSs as it is now easier to evaluate them with certainty.

## Background

Phylogenetic tree reconstruction from molecular sequences (PTMS) was first suggested by Emile Zuckerkandl and Linus Pauling [1] and is now one of the major tools in the arsenal of bioinformatics. By PTMS we will understand methods which build a phylogenetic tree based solely on sequences, either coding DNA or amino acids. Of the many people who have contributed to this field, J. Felsenstein deserves special mention for his many contributions summarized in his book [2].

Computing phylogenies is ubiquitous, and not only of academic interest, but also quite practical: selecting model organisms [3], tracing disease [4], finding vectors [5], finding suitable defenses to new viruses [6], maximizing diversity for species conservation, [7] tracing ancestry and population movements [8,9] and many other problems are solved with the aid of good phylogenetic trees.

The state of testing of PTMS is far from satisfactory. This is obvious when we see the discrepancies between the results from bioinformatics and the accepted taxonomies produced by biologists, and the high confidence measures that bioinformatics has tried to attach to their results [10-12]. In short, in our experience, the distrust that biologists may have on PTMS is justifiable.

Most results in the literature supporting PTMSs use:(i) extensive simulations, (ii)measures of quality, (iii) small scale comparisons of some specific trees, (iv) some intuition. These techniques are useful, but limited. Specifically, simulations are excellent to discover errors and to find the variability that we may expect from the methods. Yet simulations usually rely on a model of evolution (e.g. Markovian evolution). It is then expected that a method which uses the same model will perform best. Measures of quality include bootstrapping, branch support confidence and indices on trees (like least squares error in distance trees or likelihood in maximum likelihood (ML) trees). These measures also rely on some statistical model which is essentially an approximation of reality. Bootstrapping values have suffered from over-confidence and/or misinterpreted and are sensitive to model violations [13-16]. Furthermore these techniques are directed towards assessing a particular tree rather than assessing the methods. Small scale comparisons are valuable but usually lack the sample size to make the results statistically strong. We consider any evidence which is in numbers less than 100 to be “anecdotal”. Any study where a subset of cases is selected is a candidate to suffer from the bias arising from an author trying to show the best examples for his/her method. Finally, intuitions are very valuable, but cannot stand scientific scrutiny. We refer as intuitions, decisions which are not based on strict optimality criteria. E.g. character weights in traditional parsimony methods; using global or local alignments; various methods for MSA computation; various measures of distances, etc.

The main problem is that there is no “gold-standard” against which methods can be evaluated. Hopefully this paper will provide two such standards.

Computing phylogenetic trees consumes millions of hours in computers around the world. Because some of these computations are so expensive and not reliable, biologists are tempted to use faster, lower quality, methods. This evaluation (which itself consumed hundreds of thousands of hours) will help bioinformaticians extract the most of their computations. In particular, as we show, some of the best PTMS are remarkably fast to compute.

We measure the quality of the PTMS in two ways, by their average difference on trees which have followed the same evolution and by their average distance to taxonomic trees. This allows us to find the best methods, and by averaging in different ways, the best components of the methods.

There is no single method that is best in all circumstances. Some of the classes of species show a preference for a particular method. This should not come as a surprise, different organisms may leave different molecular imprints of their evolution.

## Results

We now introduce the two measures on PTMSs.

### The Intra measure

**Figure 1:**
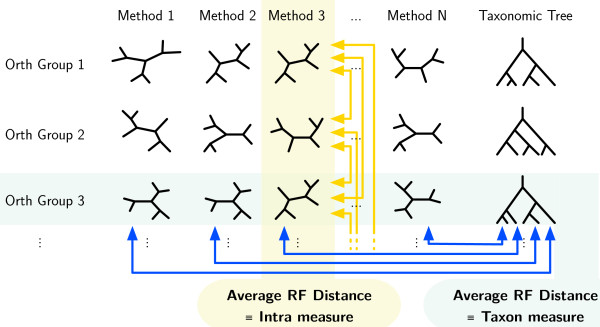
The process of computation of the Intra measure (shaded yellow, vertical) and the Taxon measure (shaded blue, horizontal).

**Figure 2:**
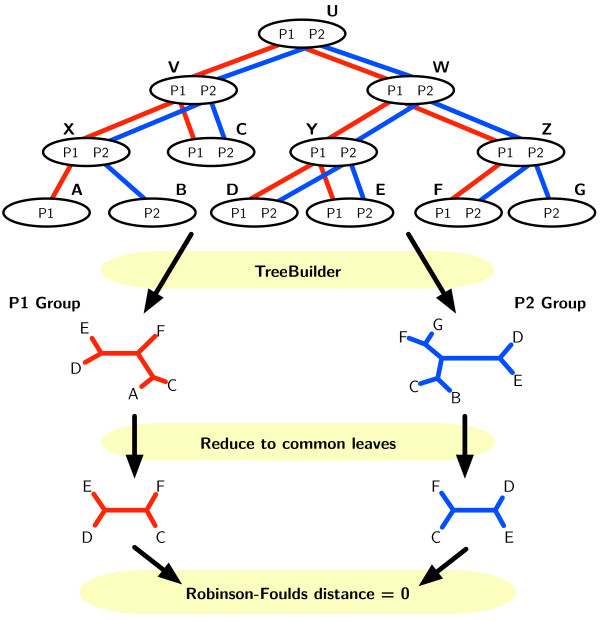
An example of the evolution of several species recovered by two proteins and the basis for the Intra measure.

For a given PTMS and several orthologous groups (OGs) we can construct a tree for every OG. The trees should all follow the same evolutionary history, hence the trees should all be compatible (Figure 1, shaded yellow). The average distance between trees built from different OGs is thus a measure of quality of the method (the smaller the distance, the better the method). We call this measure the Intra measure. Since the PTMS does not get any information about the species of the input sequences, the only way for it to produce a smaller distance between trees is by extracting information from the sequences. In this sense, the best algorithm is the algorithm which extracts the most relevant information from the sequences to derive the phylogeny; which is exactly what we want. In mathematical terms the Intra measure of a PTMS *M* is the expected value:

(1) $$\mathrm{Intra}(M)=E\left[d\left(M\left(g_{i}\right),M\left(g_{j}\right)\right)\right]$$

where *g*_*i*_ and *g*_*j*_ are two different orthologous groups. The distance *d*(.,.) is the Robinson-Foulds distance [17] between two trees built with the same PTMS over different OGs. It is computed only over the species appearing in both OGs (Figure 2). We estimate this expected value from all the available pairs of OGs. The measure will be incorrect for the cases of lateral gene transfers (LGT), where sequences do not follow the same evolution. LGT events will be few and since all methods will be affected we do not expect a bias from them.

### The Taxon measure

This measures how far the computed tree is from the true taxonomic tree. A smaller distance, averaged over a large number of OGs, means a better method. For a given PTMS and several orthologous groups (OGs) we compute the distance between the tree built on each OG and the true taxonomic tree (or its approximation from NCBI, Figure 1, shaded blue). We call this average distance the Taxon measure. The trees derived from the taxonomy represent the consensus and summary of many scientific papers, databases and experts and could be described as the “state of the art”. Errors in the taxonomy should affect all methods equally and will be like random noise.(Biases derived from the use of these methods for building the Taxonomy are discussed in the Caveats (iv) section.) In mathematical terms the Taxon measure of a PTMS *M* is the expected value:

(2) $$\mathrm{Taxon}(M)=E\left[d\left(M(g),T_{g}\right)\right]$$

where *d*(.,.) is the RF distance between two trees, *g* is an orthologous group, *M*(*g*) is the tree produced by *M* applied to the sequences in *g* and *T*_*g*_ is the taxonomic tree for the species in the group *g*. We estimate the expected value by the average over all the orthologous groups available to us. Notice, that while the taxonomic tree is a single tree, we will be sampling tens of thousands of different subsets of this single tree (and many hundreds of totally independent subsets). See Methods, Table 1, for full results. In [18,19] a similar idea is used, that of comparing the trees against a small, indisputable, topology.

**Table 1:** Taxon and Intra measure, output 1

**Average absolute/relative Taxon/Intra distances per method for non-Metazoa**					
**133525 orthologous groups**					
**Method**	**Taxon RF**		**Taxon 0-1**	**Intra**	
Mafft_InducDist_BioME	1.4254	16.75%	0.4631	1.6124	29.23%
Mafft_InducDist_FastME	1.4258	16.76%	0.4632	1.6137	29.25%
ClustalO_InducDist_BioME	1.4262	16.77%	0.4634	1.6137	29.25%
ClustalO_InducDist_FastME	1.4266	16.77%	0.4634	1.6147	29.26%
ClustalW_InducDist_BioME	1.4277	16.78%	0.4637	1.6147	29.31%
ClustalW_InducDist_FastME	1.4278	16.78%	0.4638	1.6156	29.32%
Probcons_InducDist_BioME	1.4286	16.80%	0.4647	1.6164	29.31%
Probcons_InducDist_FastME	1.4288	16.81%	0.4648	1.6178	29.32%
Poa_InducDist_BioME	1.4298	16.86%	0.4654	1.6166	29.38%
Mafft_InducDist_BioNJ	1.4307	16.74%	0.4630	1.6240	29.37%
Poa_InducDist_FastME	1.4311	16.88%	0.4656	1.6178	29.39%
Prank_InducDist_BioME	1.4314	16.81%	0.4643	1.6173	29.27%
Prank_InducDist_FastME	1.4315	16.82%	0.4644	1.6177	29.28%
ClustalO_InducDist_BioNJ	1.4318	16.76%	0.4632	1.6240	29.36%
Probcons_InducDist_BioNJ	1.4336	16.79%	0.4646	1.6266	29.40%
Prograph1_InducDist_FastME	1.4338	16.90%	0.4659	1.6211	29.40%
Prograph1_InducDist_BioME	1.4340	16.89%	0.4658	1.6206	29.39%
ClustalW_InducDist_BioNJ	1.4342	16.77%	0.4638	1.6245	29.38%
Poa_InducDist_BioNJ	1.4348	16.84%	0.4651	1.6251	29.41%
Prank_InducDist_BioNJ	1.4367	16.80%	0.4641	1.6264	29.35%
Prograph1_InducDist_BioNJ	1.4408	16.89%	0.4659	1.6326	29.54%
Probabilistic_InducDist_BioME	1.4416	16.94%	0.4670	1.6317	29.65%
Probabilistic_InducDist_FastME	1.4420	16.94%	0.4670	1.6325	29.65%
ClustalW_InducDist_LST	1.4430	16.80%	0.4643	1.6317	29.41%
Global_BioME	1.4435	17.01%	0.4682	1.6373	29.82%
GlobalWAG_BioME	1.4435	17.02%	0.4687	1.6372	29.84%
Global_FastME	1.4436	17.01%	0.4682	1.6388	29.83%
GlobalWAG_FastME	1.4439	17.03%	0.4688	1.6388	29.86%
Prank_PrankGuide	1.4444	16.76%	0.4625	1.6272	29.17%
Mafft_InducDist_LST	1.4450	16.78%	0.4635	1.6340	29.39%
ClustalO_InducDist_LST	1.4454	16.79%	0.4635	1.6342	29.40%
Prank_InducDist_LST	1.4459	16.83%	0.4645	1.6337	29.37%
GlobalJTT_BioME	1.4465	17.07%	0.4694	1.6429	29.99%
Probcons_InducDist_LST	1.4468	16.83%	0.4649	1.6361	29.43%
Poa_InducDist_LST	1.4468	16.88%	0.4655	1.6341	29.46%
GlobalJTT_FastME	1.4473	17.07%	0.4693	1.6441	30.01%
Global_BioNJ	1.4474	16.99%	0.4678	1.6454	29.86%
LogDelGlobal_BioME	1.4474	17.13%	0.4708	1.6448	30.17%
GlobalWAG_BioNJ	1.4480	17.00%	0.4683	1.6445	29.86%
ClustalO_CodonDist_BioME	1.4481	16.80%	0.4650	1.6571	30.04%
ClustalW_CodonDist_BioME	1.4481	16.81%	0.4653	1.6584	30.10%
ClustalO_CodonDist_FastME	1.4483	16.81%	0.4653	1.6588	30.06%
LogDelGlobal_FastME	1.4483	17.14%	0.4710	1.6464	30.19%
Mafft_CodonDist_FastME	1.4485	16.80%	0.4649	1.6580	30.04%
PartialOrder_InducDist_FastME	1.4488	17.32%	0.4736	1.6414	30.18%
PartialOrder_InducDist_BioME	1.4489	17.31%	0.4734	1.6408	30.17%
Probabilistic_InducDist_BioNJ	1.4489	16.94%	0.4671	1.6444	29.78%
Mafft_CodonDist_BioME	1.4490	16.80%	0.4649	1.6569	30.03%
GlobalLG_BioME	1.4490	17.10%	0.4700	1.6490	30.13%
Global_NJ	1.4490	17.02%	0.4683	1.6483	29.94%
ClustalW_CodonDist_FastME	1.4492	16.82%	0.4654	1.6602	30.12%
Prograph1_InducDist_LST	1.4503	16.92%	0.4660	1.6397	29.56%
GlobalLG_FastME	1.4504	17.11%	0.4704	1.6505	30.14%
Probcons_CodonDist_BioME	1.4505	16.83%	0.4660	1.6598	30.11%
GlobalJTT_BioNJ	1.4507	17.05%	0.4693	1.6496	29.99%
Probcons_CodonDist_FastME	1.4509	16.83%	0.4659	1.6615	30.13%
Poa_CodonDist_FastME	1.4513	16.89%	0.4669	1.6624	30.18%
Poa_CodonDist_BioME	1.4514	16.89%	0.4671	1.6608	30.16%
LogDelGlobal_BioNJ	1.4518	17.12%	0.4709	1.6525	30.21%
Prank_CodonDist_FastME	1.4534	16.88%	0.4664	1.6605	30.04%
Prank_CodonDist_BioME	1.4538	16.87%	0.4663	1.6592	30.02%
PartialOrder_InducDist_BioNJ	1.4538	17.29%	0.4731	1.6478	30.18%
GlobalLG_BioNJ	1.4543	17.08%	0.4696	1.6571	30.15%
Prograph1_CodonDist_BioME	1.4580	16.94%	0.4678	1.6625	30.11%
Prograph1_CodonDist_FastME	1.4583	16.94%	0.4679	1.6642	30.12%
Probabilistic_InducDist_LST	1.4584	16.96%	0.4672	1.6511	29.77%
GlobalWAG_LST	1.4593	17.02%	0.4685	1.6526	29.86%
Global_LST	1.4595	17.01%	0.4682	1.6532	29.87%
PartialOrder_InducDist_LST	1.4609	17.28%	0.4730	1.6524	30.13%
GlobalJTT_LST	1.4623	17.07%	0.4694	1.6570	29.99%
Mafft_CodonDist_BioNJ	1.4625	16.83%	0.4651	1.6723	30.15%
Probabilistic_CodonDist_FastME	1.4626	16.99%	0.4691	1.6753	30.42%
ClustalO_CodonDist_BioNJ	1.4629	16.84%	0.4653	1.6742	30.20%
Probabilistic_CodonDist_BioME	1.4630	16.98%	0.4690	1.6742	30.40%
LogDelGlobal_LST	1.4632	17.13%	0.4705	1.6594	30.19%
ClustalW_CodonDist_BioNJ	1.4634	16.84%	0.4653	1.6761	30.24%
Probcons_CodonDist_BioNJ	1.4642	16.85%	0.4659	1.6754	30.25%
GlobalLG_LST	1.4647	17.10%	0.4700	1.6630	30.13%
Poa_CodonDist_BioNJ	1.4654	16.93%	0.4673	1.6777	30.30%
ClustalW_CodonDist_LST	1.4671	16.82%	0.4653	1.6623	30.02%
Prank_CodonDist_BioNJ	1.4679	16.89%	0.4668	1.6761	30.18%
Mafft_CodonDist_LST	1.4705	16.82%	0.4649	1.6620	29.96%
ClustalO_CodonDist_LST	1.4705	16.85%	0.4657	1.6631	30.00%
Prograph1_CodonDist_BioNJ	1.4717	16.95%	0.4676	1.6798	30.26%
Probcons_CodonDist_LST	1.4728	16.85%	0.4660	1.6654	30.05%
Poa_CodonDist_LST	1.4730	16.94%	0.4676	1.6656	30.10%
Prank_PhyML	1.4733	17.30%	0.4738	1.7164	31.22%
Local_BioME	1.4738	17.32%	0.4733	1.6832	30.69%
Prank_CodonDist_LST	1.4739	16.88%	0.4663	1.6636	29.96%
LocalWAG_BioME	1.4748	17.35%	0.4739	1.6850	30.74%
Local_FastME	1.4750	17.34%	0.4736	1.6851	30.71%
LocalWAG_FastME	1.4753	17.36%	0.4739	1.6865	30.76%
PartialOrder_CodonDist_BioME	1.4753	17.42%	0.4773	1.6835	30.95%
PartialOrder_CodonDist_FastME	1.4763	17.43%	0.4773	1.6850	30.98%
Prank_RAxMLG	1.4780	17.34%	0.4744	1.7235	31.30%
Probabilistic_CodonDist_BioNJ	1.4781	17.00%	0.4692	1.6935	30.57%
Prograph1_CodonDist_LST	1.4782	16.94%	0.4676	1.6682	30.07%
Local_BioNJ	1.4785	17.31%	0.4733	1.6903	30.74%
LocalWAG_BioNJ	1.4799	17.35%	0.4739	1.6928	30.80%
Mafft_PhyML	1.4804	17.49%	0.4767	1.7262	31.56%
Local_NJ	1.4806	17.33%	0.4735	1.6930	30.78%
ClustalO_PhyML	1.4809	17.49%	0.4778	1.7271	31.62%
GlobalCodonPAM_BioME	1.4815	17.17%	0.4725	1.6941	30.87%
GlobalCodonPAM_FastME	1.4821	17.16%	0.4724	1.6956	30.89%
ClustalW_PhyML	1.4823	17.55%	0.4790	1.7292	31.69%
Prograph1_PhyML	1.4828	17.50%	0.4771	1.7259	31.49%
Poa_PhyML	1.4832	17.54%	0.4783	1.7297	31.63%
Probcons_PhyML	1.4837	17.56%	0.4788	1.7296	31.70%
LogDelLocal_BioME	1.4837	17.55%	0.4774	1.6970	31.16%
Probabilistic_CodonDist_LST	1.4843	17.01%	0.4689	1.6812	30.38%
LogDelLocal_FastME	1.4856	17.55%	0.4775	1.6987	31.18%
LocalLG_BioME	1.4859	17.53%	0.4768	1.7014	31.13%
Mafft_RAxMLG	1.4862	17.53%	0.4773	1.7361	31.67%
LocalLG_FastME	1.4866	17.54%	0.4770	1.7023	31.14%
LocalJTT_BioME	1.4872	17.57%	0.4776	1.6980	31.04%
Local_LST	1.4879	17.31%	0.4731	1.6956	30.70%
Prograph1_RAxMLG	1.4880	17.54%	0.4778	1.7351	31.59%
ClustalO_RAxMLG	1.4882	17.55%	0.4782	1.7371	31.72%
LocalWAG_LST	1.4883	17.34%	0.4733	1.6966	30.74%
Probabilistic_PhyML	1.4885	17.63%	0.4802	1.7342	31.77%
PartialOrder_CodonDist_BioNJ	1.4885	17.44%	0.4770	1.7003	31.06%
LocalJTT_FastME	1.4887	17.58%	0.4776	1.6998	31.06%
Poa_RAxMLG	1.4889	17.58%	0.4789	1.7402	31.74%
ClustalW_RAxMLG	1.4894	17.62%	0.4796	1.7392	31.79%
LogDelLocal_BioNJ	1.4895	17.56%	0.4774	1.7034	31.19%
ClustalO_RAxML	1.4897	17.80%	0.4823	1.7384	31.95%
Probcons_RAxMLG	1.4901	17.60%	0.4791	1.7408	31.82%
ClustalW_RAxML	1.4904	17.83%	0.4832	1.7400	32.00%
LocalLG_BioNJ	1.4917	17.53%	0.4771	1.7085	31.15%
PartialOrder_CodonDist_LST	1.4918	17.41%	0.4765	1.6844	30.78%
LocalJTT_BioNJ	1.4927	17.56%	0.4774	1.7053	31.07%
Probcons_RAxML	1.4929	17.89%	0.4836	1.7420	32.07%
Prank_Parsimony	1.4934	17.36%	0.4730	1.7379	31.34%
Poa_RAxML	1.4939	17.85%	0.4830	1.7441	32.07%
Prograph1_RAxML	1.4942	17.82%	0.4821	1.7412	31.95%
LogDelLocal_LST	1.4944	17.50%	0.4765	1.7047	31.06%
Prograph1_Parsimony	1.4948	17.40%	0.4742	1.7366	31.30%
Probabilistic_RAxMLG	1.4950	17.67%	0.4804	1.7439	31.86%
GlobalCodonPAM_BioNJ	1.4951	17.18%	0.4723	1.7056	30.91%
Prank_RAxML	1.4956	18.17%	0.4878	1.7357	31.96%
LocalLG_LST	1.4973	17.48%	0.4762	1.7086	31.04%
LocalJTT_LST	1.4986	17.52%	0.4765	1.7063	30.96%
Mafft_RAxML	1.5006	18.30%	0.4898	1.7437	32.22%
GlobalCodonPAM_LST	1.5022	17.16%	0.4717	1.6930	30.68%
Probabilistic_RAxML	1.5074	18.40%	0.4921	1.7490	32.32%
ClustalW_Parsimony	1.5105	17.63%	0.4786	1.7603	31.90%
Probabilistic_Parsimony	1.5109	17.56%	0.4770	1.7618	31.88%
PartialOrder_PhyML	1.5153	18.37%	0.4923	1.7581	32.53%
Mafft_Parsimony	1.5220	17.75%	0.4804	1.7713	32.10%
ClustalO_Parsimony	1.5226	17.75%	0.4803	1.7728	32.12%
PartialOrder_RAxMLG	1.5231	18.38%	0.4923	1.7681	32.61%
Poa_Parsimony	1.5257	17.87%	0.4823	1.7779	32.28%
Probcons_Parsimony	1.5301	17.84%	0.4823	1.7823	32.32%
PartialOrder_RAxML	1.5374	19.12%	0.5039	1.7771	33.18%
PartialOrder_Parsimony	1.5543	18.08%	0.4873	1.8205	32.79%
LocalCodonPAM_LST	1.5741	18.29%	0.4886	1.7544	32.02%
LocalCodonPAM_BioME	1.5749	18.54%	0.4916	1.7766	32.63%
LocalCodonPAM_FastME	1.5749	18.54%	0.4917	1.7788	32.66%
LocalCodonPAM_BioNJ	1.5913	18.60%	0.4924	1.7853	32.58%
Prograph1_Gap	1.9433	21.39%	0.5260	2.4423	45.15%
Prank_Gap	1.9785	21.75%	0.5299	2.4897	46.18%
Poa_Gap	2.3287	25.84%	0.5777	2.7803	52.61%
Probabilistic_Gap	2.3428	26.34%	0.5818	2.7972	53.64%
PartialOrder_Gap	2.4197	24.94%	0.5591	2.8379	51.69%
ClustalW_Gap	2.5021	27.89%	0.5982	2.9157	56.10%
Mafft_Gap	2.5251	27.01%	0.5850	2.9296	55.42%
ClustalO_Gap	2.5572	27.63%	0.5921	2.9586	56.38%
Probcons_Gap	2.5816	28.39%	0.6012	2.9752	57.11%
GlobalSynPAM_LST	2.6621	30.23%	0.6191	2.5864	48.01%
LocalSynPAM_LST	2.6831	30.51%	0.6209	2.6048	48.36%
GlobalSynPAM_BioNJ	2.8077	30.97%	0.6218	2.8184	51.99%
LocalSynPAM_BioNJ	2.8226	31.21%	0.6238	2.8305	52.25%
GlobalSynPAM_BioME	2.8493	31.80%	0.6272	2.8923	54.71%
GlobalSynPAM_FastME	2.8601	31.88%	0.6276	2.9101	55.07%
LocalSynPAM_BioME	2.8607	31.97%	0.6285	2.9007	54.80%
LocalSynPAM_FastME	2.8773	32.07%	0.6289	2.9244	55.25%
Method	Taxon RF		Taxon 0-1	Intra	
PartialOrder_CodonDist_BioNJ	2.2812	20.25%	0.5524	3.1378	39.11%
PartialOrder_CodonDist_BioME	2.2940	20.43%	0.5551	3.1490	39.43%
Prank_CodonDist_BioNJ	2.2949	20.18%	0.5500	3.1932	39.45%
PartialOrder_CodonDist_FastME	2.3042	20.47%	0.5554	3.1604	39.52%
PartialOrder_CodonDist_LST	2.3069	20.24%	0.5509	3.1061	38.65%
Prank_CodonDist_BioME	2.3103	20.37%	0.5533	3.2056	39.60%
Prank_CodonDist_LST	2.3123	20.15%	0.5492	3.1532	38.89%
GlobalCodonPAM_BioNJ	2.3155	20.47%	0.5545	3.1970	39.76%
Prank_CodonDist_FastME	2.3202	20.40%	0.5532	3.2164	39.67%
GlobalCodonPAM_BioME	2.3320	20.67%	0.5565	3.2058	40.05%
Prograph1_CodonDist_BioNJ	2.3362	20.19%	0.5491	3.2380	39.58%
GlobalCodonPAM_LST	2.3393	20.42%	0.5521	3.1599	39.25%
GlobalCodonPAM_FastME	2.3445	20.73%	0.5566	3.2180	40.16%
Prograph1_CodonDist_BioME	2.3482	20.35%	0.5514	3.2457	39.71%
Prograph1_CodonDist_FastME	2.3570	20.38%	0.5512	3.2527	39.76%
Prograph1_CodonDist_LST	2.3604	20.19%	0.5477	3.2084	39.14%
LocalCodonPAM_BioNJ	2.3634	20.89%	0.5579	3.2110	40.02%
LocalCodonPAM_BioME	2.3766	21.07%	0.5604	3.2148	40.23%
LocalCodonPAM_LST	2.3785	20.78%	0.5559	3.1685	39.43%
Mafft_CodonDist_BioNJ	2.3811	21.00%	0.5577	3.3489	41.02%
Probabilistic_CodonDist_BioNJ	2.3833	20.91%	0.5558	3.3162	40.94%
LocalCodonPAM_FastME	2.3873	21.12%	0.5605	3.2262	40.33%
Mafft_CodonDist_LST	2.3935	20.94%	0.5563	3.3051	40.47%
Mafft_CodonDist_BioME	2.4052	21.21%	0.5600	3.3661	41.25%
Probabilistic_CodonDist_BioME	2.4062	21.12%	0.5582	3.3381	41.24%
Probabilistic_CodonDist_LST	2.4081	20.89%	0.5546	3.2916	40.52%
Probcons_CodonDist_BioNJ	2.4086	21.00%	0.5561	3.3803	41.23%
Mafft_CodonDist_FastME	2.4146	21.24%	0.5598	3.3771	41.33%
Probabilistic_CodonDist_FastME	2.4165	21.16%	0.5583	3.3514	41.34%
Probcons_CodonDist_LST	2.4188	20.93%	0.5549	3.3364	40.70%
ClustalO_CodonDist_BioNJ	2.4205	21.26%	0.5583	3.4025	41.60%
ClustalO_CodonDist_LST	2.4294	21.16%	0.5567	3.3563	41.01%
Probcons_CodonDist_BioME	2.4393	21.24%	0.5593	3.4079	41.57%
ClustalO_CodonDist_BioME	2.4480	21.52%	0.5611	3.4249	41.86%
Probcons_CodonDist_FastME	2.4500	21.26%	0.5591	3.4202	41.65%
ClustalO_CodonDist_FastME	2.4595	21.56%	0.5611	3.4370	41.95%
Poa_CodonDist_BioNJ	2.4992	21.91%	0.5629	3.4742	42.31%
Poa_CodonDist_LST	2.5005	21.85%	0.5617	3.4225	41.76%
Poa_CodonDist_BioME	2.5280	22.17%	0.5653	3.5039	42.68%
Poa_CodonDist_FastME	2.5356	22.20%	0.5651	3.5138	42.76%
Prank_RAxMLG	2.5551	21.40%	0.5417	3.8823	45.94%
Prank_PhyML	2.5564	21.40%	0.5424	3.8813	45.93%
Prank_RAxML	2.5871	22.23%	0.5539	3.9048	46.68%
Mafft_PhyML	2.6215	22.03%	0.5475	3.9799	47.06%
Mafft_RAxMLG	2.6283	22.05%	0.5468	3.9867	47.14%
ClustalW_CodonDist_BioNJ	2.6422	22.47%	0.5631	3.7195	44.12%
Probabilistic_PhyML	2.6445	22.01%	0.5471	3.9893	47.09%
PartialOrder_PhyML	2.6466	22.06%	0.5491	3.9623	47.03%
Mafft_RAxML	2.6470	22.74%	0.5565	3.9980	47.78%
Probabilistic_RAxMLG	2.6494	21.98%	0.5465	3.9974	47.17%
Probcons_PhyML	2.6509	22.16%	0.5482	4.0128	47.39%
ClustalW_CodonDist_LST	2.6516	22.41%	0.5623	3.6854	43.66%
PartialOrder_RAxMLG	2.6543	22.10%	0.5492	3.9743	47.17%
Prograph1_PhyML	2.6589	21.77%	0.5434	4.0053	46.91%
Prograph1_RAxMLG	2.6618	21.76%	0.5435	4.0155	47.02%
Probabilistic_RAxML	2.6641	22.60%	0.5558	4.0059	47.75%
Probcons_RAxMLG	2.6653	22.25%	0.5477	4.0258	47.53%
ClustalO_PhyML	2.6661	22.44%	0.5504	4.0345	47.66%
ClustalW_CodonDist_BioME	2.6729	22.74%	0.5662	3.7448	44.39%
Probcons_RAxML	2.6758	22.82%	0.5563	4.0312	48.04%
PartialOrder_RAxML	2.6764	22.87%	0.5594	3.9887	47.83%
ClustalO_RAxMLG	2.6768	22.45%	0.5497	4.0466	47.81%
ClustalW_CodonDist_FastME	2.6829	22.78%	0.5662	3.7554	44.47%
Prograph1_RAxML	2.6848	22.49%	0.5534	4.0284	47.66%
ClustalO_RAxML	2.6859	22.95%	0.5578	4.0453	48.22%
Poa_PhyML	2.7723	23.26%	0.5574	4.1128	48.54%
Poa_RAxMLG	2.7870	23.33%	0.5573	4.1280	48.70%
Poa_RAxML	2.7972	23.77%	0.5641	4.1323	49.10%
PartialOrder_InducDist_BioNJ	2.8486	22.72%	0.5518	4.0863	47.74%
PartialOrder_InducDist_BioME	2.8513	22.82%	0.5532	4.0824	47.73%
PartialOrder_InducDist_LST	2.8539	22.67%	0.5509	4.0550	47.26%
PartialOrder_InducDist_FastME	2.8566	22.83%	0.5531	4.0869	47.77%
ClustalW_PhyML	2.8682	23.54%	0.5572	4.2573	49.54%
Prank_InducDist_LST	2.8697	22.53%	0.5476	4.0833	47.19%
Prank_InducDist_BioME	2.8739	22.66%	0.5492	4.1142	47.54%
Prank_InducDist_BioNJ	2.8750	22.63%	0.5485	4.1233	47.68%
Prank_InducDist_FastME	2.8788	22.69%	0.5495	4.1202	47.60%
ClustalW_RAxMLG	2.8821	23.54%	0.5562	4.2710	49.65%
Prank_PrankGuide	2.8831	22.46%	0.5464	4.0804	47.16%
ClustalW_RAxML	2.8915	23.90%	0.5613	4.2771	50.04%
Prograph1_InducDist_BioME	2.9224	22.65%	0.5473	4.1828	47.96%
Prograph1_InducDist_BioNJ	2.9236	22.62%	0.5466	4.1902	48.12%
GlobalJTT_LST	2.9260	23.21%	0.5547	4.1389	48.32%
Prograph1_InducDist_FastME	2.9286	22.66%	0.5473	4.1872	47.99%
GlobalJTT_BioNJ	2.9316	23.34%	0.5552	4.1770	48.81%
Prograph1_InducDist_LST	2.9324	22.57%	0.5458	4.1685	47.77%
GlobalJTT_BioME	2.9341	23.43%	0.5567	4.1737	48.83%
GlobalWAG_BioNJ	2.9416	23.34%	0.5544	4.1848	48.88%
GlobalWAG_LST	2.9424	23.27%	0.5543	4.1488	48.39%
GlobalJTT_FastME	2.9441	23.48%	0.5571	4.1841	48.92%
GlobalWAG_BioME	2.9444	23.44%	0.5562	4.1813	48.84%
GlobalLG_LST	2.9502	23.48%	0.5577	4.1604	48.68%
Global_BioNJ	2.9526	23.48%	0.5563	4.1963	48.99%
Global_LST	2.9530	23.38%	0.5550	4.1574	48.50%
GlobalWAG_FastME	2.9560	23.49%	0.5565	4.1937	48.95%
Global_BioME	2.9562	23.54%	0.5570	4.1932	48.98%
GlobalLG_BioNJ	2.9566	23.58%	0.5580	4.2009	49.19%
Global_FastME	2.9651	23.57%	0.5570	4.2033	49.08%
GlobalLG_BioME	2.9675	23.71%	0.5600	4.2011	49.22%
GlobalLG_FastME	2.9754	23.73%	0.5600	4.2117	49.32%
Global_NJ	2.9831	23.65%	0.5571	4.2241	49.39%
Mafft_InducDist_LST	2.9880	23.41%	0.5536	4.2495	48.94%
LocalJTT_LST	2.9926	23.63%	0.5582	4.1898	48.96%
Mafft_InducDist_BioNJ	2.9979	23.51%	0.5538	4.2970	49.46%
LogDelGlobal_BioME	3.0003	23.90%	0.5606	4.2157	49.62%
Mafft_InducDist_BioME	3.0031	23.58%	0.5549	4.2901	49.32%
LogDelGlobal_LST	3.0041	23.74%	0.5594	4.1930	49.20%
Probabilistic_InducDist_BioME	3.0041	23.75%	0.5574	4.2610	49.52%
Probabilistic_InducDist_BioNJ	3.0045	23.69%	0.5561	4.2621	49.55%
LogDelGlobal_FastME	3.0052	23.91%	0.5606	4.2223	49.67%
Probabilistic_InducDist_FastME	3.0086	23.75%	0.5573	4.2673	49.56%
LocalWAG_LST	3.0094	23.66%	0.5582	4.2019	49.06%
Probabilistic_InducDist_LST	3.0094	23.63%	0.5561	4.2414	49.22%
Mafft_InducDist_FastME	3.0124	23.60%	0.5547	4.2986	49.39%
LogDelGlobal_BioNJ	3.0127	23.83%	0.5593	4.2454	49.82%
LocalJTT_BioNJ	3.0132	23.82%	0.5595	4.2476	49.65%
LocalJTT_BioME	3.0143	23.87%	0.5601	4.2352	49.53%
Local_LST	3.0154	23.67%	0.5572	4.2069	49.07%
LocalLG_LST	3.0189	23.81%	0.5597	4.2167	49.33%
LocalWAG_BioME	3.0214	23.88%	0.5598	4.2421	49.55%
LocalJTT_FastME	3.0218	23.91%	0.5604	4.2426	49.60%
LocalWAG_BioNJ	3.0260	23.83%	0.5588	4.2574	49.71%
Probcons_InducDist_LST	3.0276	23.47%	0.5530	4.2897	49.24%
Local_BioME	3.0277	23.87%	0.5588	4.2485	49.61%
Local_BioNJ	3.0294	23.81%	0.5582	4.2636	49.75%
LocalWAG_FastME	3.0294	23.90%	0.5598	4.2506	49.63%
Local_FastME	3.0370	23.92%	0.5592	4.2565	49.67%
LocalLG_BioME	3.0403	24.10%	0.5619	4.2607	49.91%
LocalLG_BioNJ	3.0424	24.00%	0.5606	4.2776	50.06%
Probcons_InducDist_BioNJ	3.0435	23.58%	0.5530	4.3357	49.75%
LogDelLocal_LST	3.0466	24.00%	0.5606	4.2283	49.59%
Probcons_InducDist_BioME	3.0490	23.66%	0.5542	4.3342	49.70%
Local_NJ	3.0493	23.95%	0.5591	4.2748	49.97%
LogDelLocal_BioME	3.0518	24.18%	0.5620	4.2584	50.05%
ClustalO_InducDist_LST	3.0520	23.70%	0.5546	4.3168	49.59%
LocalLG_FastME	3.0524	24.14%	0.5619	4.2721	50.00%
Probcons_InducDist_FastME	3.0593	23.71%	0.5544	4.3447	49.80%
ClustalO_InducDist_BioNJ	3.0623	23.82%	0.5552	4.3603	50.07%
LogDelLocal_FastME	3.0628	24.22%	0.5621	4.2679	50.13%
ClustalO_InducDist_BioME	3.0674	23.90%	0.5565	4.3558	50.01%
LogDelLocal_BioNJ	3.0684	24.16%	0.5610	4.2913	50.31%
ClustalO_InducDist_FastME	3.0739	23.94%	0.5567	4.3618	50.06%
Poa_InducDist_LST	3.1107	24.27%	0.5591	4.3594	50.08%
Poa_InducDist_BioNJ	3.1240	24.38%	0.5593	4.3979	50.51%
Poa_InducDist_BioME	3.1344	24.49%	0.5605	4.4035	50.54%
Poa_InducDist_FastME	3.1409	24.51%	0.5607	4.4117	50.62%
ClustalW_InducDist_LST	3.2044	24.57%	0.5585	4.5147	51.29%
ClustalW_InducDist_BioNJ	3.2100	24.69%	0.5597	4.5406	51.66%
ClustalW_InducDist_BioME	3.2189	24.79%	0.5609	4.5416	51.61%
ClustalW_InducDist_FastME	3.2252	24.80%	0.5608	4.5485	51.67%
GlobalSynPAM_LST	3.5173	31.10%	0.6338	3.9687	50.34%
LocalSynPAM_LST	3.5271	31.11%	0.6334	3.9732	50.35%
Prograph1_Parsimony	3.6661	27.24%	0.5749	4.8664	55.21%
GlobalSynPAM_BioNJ	3.7288	31.89%	0.6350	4.1870	52.87%
LocalSynPAM_BioNJ	3.7325	31.85%	0.6342	4.1860	52.82%
LocalSynPAM_BioME	3.9135	32.87%	0.6370	4.4534	55.73%
GlobalSynPAM_BioME	3.9186	32.95%	0.6384	4.4700	56.00%
LocalSynPAM_FastME	3.9554	33.01%	0.6372	4.4974	56.11%
GlobalSynPAM_FastME	3.9586	33.08%	0.6386	4.5120	56.32%
Prank_Parsimony	4.1293	30.16%	0.5895	5.2537	59.34%
Probabilistic_Parsimony	4.2511	31.26%	0.5968	5.3638	60.93%
ClustalW_Parsimony	4.2646	31.42%	0.5993	5.3851	61.15%
Mafft_Parsimony	4.3086	31.86%	0.6027	5.3770	61.22%
ClustalO_Parsimony	4.3105	31.79%	0.6007	5.3842	61.27%
Probcons_Parsimony	4.3464	32.23%	0.6050	5.4023	61.60%
Poa_Parsimony	4.3852	32.52%	0.6067	5.4298	61.97%
PartialOrder_Parsimony	4.4223	32.34%	0.6057	5.4796	62.40%
Prograph1_Gap	4.8752	35.60%	0.6223	5.8352	67.80%
Prank_Gap	5.6787	40.79%	0.6442	6.3558	73.73%
PartialOrder_Gap	5.7425	41.19%	0.6477	6.3644	74.18%
Probabilistic_Gap	5.9593	45.15%	0.6773	6.4529	76.15%
Poa_Gap	6.2008	46.51%	0.6820	6.5492	77.39%
ClustalW_Gap	6.2522	47.55%	0.6892	6.5718	77.93%
ClustalO_Gap	6.2747	47.15%	0.6837	6.5759	77.85%
Mafft_Gap	6.2803	46.87%	0.6813	6.5867	77.88%
Probcons_Gap	6.3514	48.04%	0.6901	6.6129	78.48%

To achieve statistical significance we consider complete genomes and apply the methods to all the OGs possible (with at least 4 species) according to the OMA database [20,21]. This gives us very large sample sizes and an unbiased sample, as almost nothing is excluded (see methods for details).

To describe the PTMSs unambiguously we need to use a descriptive name for each one. The convention that we use describes the steps which are used to build the tree. For example, stands for the name of the procedure which starts by making a multiple sequence alignment (MSA) using ClustalW, then derives the distances from the pairwise alignments induced by the MSA and finally builds a tree from these distances using the BioNJ algorithm. A method is then a sequence of components which start from the molecular sequences and end with a phylogenetic tree. The components of the tree building methods used here are listed in Table 2.

**Table 2:** Classification of component methods of the PTMS (see methods for full details)

**Description**	**Methods**
Multiple sequence	ClustalO, ClustalW, Mafft, PartialOrder,
alignment	
	Poa, Prank, Probabilistic, Probcons,
	Prograph
Methods on MSAs	Gap, Parsimony, PhyML, RAxML, RAxMLG
Pairwise alignments	GlobalCodonPAM, GlobalJTT, GlobalLG,
	GlobalSynPAM, GlobalWAG, GlobalGCB,
	LocalCodonPAM, LocalJTT, LocalLG,
	LocalSynPAM, LocalWAG, LocalGCB,
	LogDelGlobal, LogDelLocal
Pairwise alignments	CodonDist, InducDist
from MSAs	
Distance methods	BioME, BioNJ, FastME, LST, NJ

Most of the possible compatible combinations were tried. Notice that the total number of methods can grow very quickly, for this study 176 PTMSs were tested.

Our main results are: the introduction of the Intra and Taxon measures to evaluate PTMSs; the excellent correlation between them; the top rated PTMSs for Metazoa and non-Metazoa; the results on best components, i.e. best MSA methods, best tree building methods and best pairwise alignment methods.

**Figure 3:**
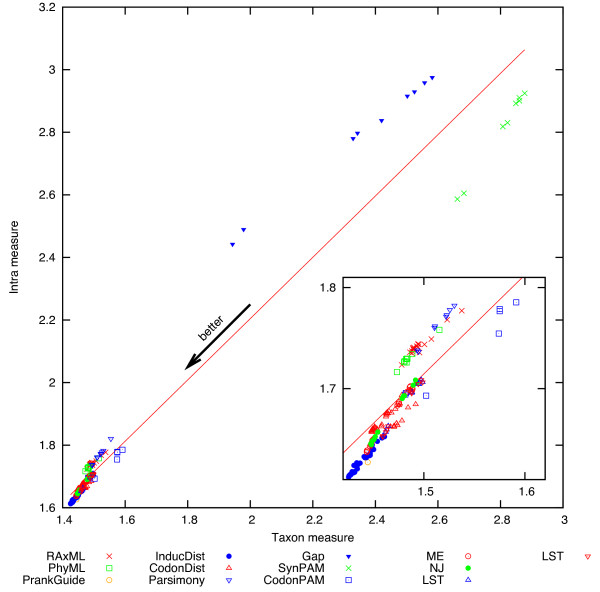
Intra(M) vs Taxon(M) measures for all methods.

Figure 3 shows a plot of the PTMSs on their Intra vs Taxon measures. It can be seen that the two measures are extremely well correlated. Table 3 shows the same correlation in numerical form and for each species class. Here a “class” means a convenient group of related species, explained in more detail in the methods section.

**Table 3:** Intra/Taxon correlation coefficients over all PTMS

**Class**	**Pearson’s**
non Metazoa	0.9771
Actinobacteria	0.9807
Archaea	0.9655
Firmicutes	0.9810
Metazoa	0.9505
OtherBacteria	0.9698
OtherEukaryota	0.9841
Proteobacteria	0.9800

It should be noted that, for a given class or set of classes, the numerical values of the Intra measure for all the PTMSs are comparable (lower values mean better methods). So are the values of the Taxon measure. But, for a given class, the Intra and Taxon measures are not numerically comparable, as they are taken over different sets, in one case over all the pairs of OGs which intersect on 4 or more leaves in the second case over all the OGs. This is why we compare the orderings (usually by computing Pearson’s correlation coefficient) of the PTMSs by each measure, but not the corresponding numerical values.

Tables 4 and 5 show the best (Taxon) scoring PTMS. The first table shows the top 3 methods for Metazoa and for non-Metazoa. The results group well in two sets. Metazoa favors codon-based methods whereas the rest favor induced distance methods. In terms of sample sizes this division is quite even, the number of OGs are in a 1:2 relation but since Metazoa has larger groups and longer sequences, the total amino acids involved are close to 1:1 (Table 6).

The symbol “≫” stands for a method which is better than another with statistical significance better than 1 in a million (p-value < 1e-6). The symbol “>” stands for a p-value < 0.05 and the symbol “≥” means its p-value > 0.05.

To justify the grouping of the classes we have computed the correlations between the classes. Table 7 shows Pearson’s correlation coefficients of the Intra measures for all the classes against each other.

**Table 4:** The top PTMS according to the Taxon measure

**Class**	**Best 3 methods**
Metazoa	PartialOrder_CodonDist_BioNJ > PartialOrder_CodonDist_BioME ≥ Prank_CodonDist_BioNJ
non Metazoa	Mafft_InducDist_BioME ≥ Mafft_InducDist_FastME ≥ ClustalO_InducDist_BioME

**Table 5:** The top PTMS according to the Taxon measure

**Class**	**Best 3 methods**
Actinobacteria	Probcons_InducDist_BioME ≥ Mafft_InducDist_BioME ≥ Prank_InducDist_BioME
Archaea	Mafft_InducDist_BioNJ ≥ Mafft_InducDist_FastME ≥ Mafft_InducDist_BioME
Firmicutes	Probcons_CodonDist_FastME ≥ Mafft_CodonDist_FastME ≥ ClustalO_CodonDist_FastME
OtherBacteria	ClustalO_InducDist_FastME ≥ Mafft_InducDist_FastME ≥ Mafft_InducDist_BioME
OtherEukaryota	ClustalO_InducDist_BioME ≥ ClustalO_InducDist_FastME ≥ ClustalO_InducDist_BioNJ
Proteobacteria	Mafft_InducDist_BioME ≥ Mafft_InducDist_FastME ≥ ClustalW_InducDist_FastME

**Table 6:** Sizes of the classes

**Class**	**species**	**OGs OMA**	**OGs kept**	**seqs**	**seqs/grp**	**aa/seq**
Actinobacteria	80	41823	18877	199556	10.57	353.74
Archaea	81	23276	11032	115290	10.45	304.97
Firmicutes	89	31599	14526	172326	11.86	323.69
Metazoa	70	149311	59613	773920	12.98	526.86
OtherBacteria	131	38073	15484	150934	9.75	362.63
OtherEukaryota	39	71641	22745	151693	6.67	498.26
Proteobacteria	265	103585	50861	692257	13.61	342.44
Total	755	459308	193138	2255976	11.68	415.19

**Table 7:** Correlation of the Intra measure of all PTMS between classes

**Class**	**Acti**	**Arch**	**Firm**	**Meta**	**OBac**	**OEuk**	**Prot**
Actinobacteria	1.000	0.970	0.979	0.574	0.978	0.986	0.995
Archaea		1.000	0.993	0.540	0.996	0.979	0.986
Firmicutes			1.000	0.599	0.994	0.984	0.993
Metazoa				1.000	0.531	0.539	0.582
OtherBacteria					1.000	0.988	0.991
OtherEukaryota						1.000	0.987
Proteobacteria							1.000

The average correlation for non-Metazoa is 0.9867 in a tight range, from 0.9696 to 0.9964. Notice also that OtherEukaryota share the same preferences for the methods as Archaea and Bacteria away from Metazoa. All the correlations with Metazoa are much lower. The natural grouping of the classes is to have one group with Metazoa and another group with the rest. The very high correlations of the different non-Metazoa classes are the main argument supporting the quality of the Taxon measure. The measure is strong enough to replicate the rankings on several groups. This is a form of bootstrapping, as the results are replicated from independent different samples.

### Averaging over the component methods

Tables 8,9,10,11,12 and 13 show results over component methods for the Taxon measure. We are working under the assumption that better trees derived from variants of the components (e.g. MSAs) mean better components (e.g. better MSAs). While this may be controversial, it is very difficult to argue the opposite, see [19]. These results are aggregations of various classes and various methods. In all cases care is taken to include the same companion methods for each comparison. The numerical value *Δ* shows the difference of the Taxon measures (and its 95% confidence margin) between the methods. It measures the average difference of RF distances or wrong splits, e.g. *Δ*=1 means that on the average one method makes one additional mistake per tree. *n* indicates the number of OGs which have been used to measure this difference (in some cases the OGs end up used more than once, for example for different MSAs when comparing ML methods). See Methods, Table 14.

**Table 8:** Ranking and differences among tree builders based on MSAs

	***Δ***	***n***
non Metazoa		
PhyML ≫ RAxMLG	-0.0063±0.0005	1201725
RAxMLG ≫ RAxML	-0.0084±0.0007	1201725
RAxML ≫ Parsimony	-0.0180±0.0010	1201725
Parsimony ≫ Gap	-0.8350±0.0044	1201725
Metazoa		
PhyML ≫ RAxMLG	-0.0083±0.0017	536517
RAxMLG ≫ RAxML	-0.0166±0.0019	536517
RAxML ≫ Parsimony	-1.5305±0.0085	536517
Parsimony ≫ Gap	-1.7256±0.0081	536517

**Table 9:** Ranking and differences among pairwise alignments

	***Δ***	***n***
non Metazoa		
Global ≫ LogDelGlobal	-0.0042±0.0009	534100
LogDelGlobal ≫ Local	-0.0261±0.0012	534100
Local ≫ LogDelLocal	-0.0095±0.0009	534100
LogDelLocal > GlobalCodonPAM	-0.0019±0.0017	534100
GlobalCodonPAM ≫ LocalCodonPAM	-0.0886±0.0015	534100
LocalCodonPAM ≫ GlobalSynPAM	-1.2160±0.0077	534100
GlobalSynPAM ≫ LocalSynPAM	-0.0161±0.0014	534100
Metazoa		
GlobalCodonPAM ≫ LocalCodonPAM	-0.0436±0.0028	238452
LocalCodonPAM ≫ Global	-0.5803±0.0074	238452
Global ≫ LogDelGlobal	-0.0488±0.0035	238452
LogDelGlobal ≫ Local	-0.0218±0.0037	238452
Local ≫ LogDelLocal	-0.0300±0.0032	238452
LogDelLocal ≫ GlobalSynPAM	-0.7234±0.0112	238452
GlobalSynPAM ≥ LocalSynPAM	-0.0013±0.0028	238452

**Table 10:** Ranking and differences among pairwise alignments derived from MSAs

	***Δ***	***n***
non Metazoa		
InducDist ≫ CodonDist	-0.0257±0.0005	4806900
Metazoa		
CodonDist ≫ InducDist	-0.5896±0.0024	2146068

**Table 11:** Ranking and differences among tree building methods based on distances

	***Δ***	***n***
non Metazoa		
BioME ≫ FastME	-0.0013±0.0002	4272800
FastME ≫ BioNJ	-0.0043±0.0004	4272800
BioNJ > NJ	-0.0018±0.0010	267050
NJ ≫ LST	-0.0089±0.0014	267050
Metazoa		
LST ≫ BioNJ	-0.0125±0.0011	1907616
BioNJ ≫ BioME	-0.0195±0.0010	1907616
BioME ≫ FastME	-0.0108±0.0006	1907616
FastME ≫ NJ	-0.0151±0.0036	119226

**Table 12:** Ranking and differences among empirical substitution matrices

	***Δ***	***n***
non Metazoa		
GCB ≥ WAG	-0.0005±0.0005	1068200
WAG ≫ JTT	-0.0076±0.0006	1068200
JTT > LG	-0.0007±0.0006	1068200
Metazoa		
JTT ≫ WAG	-0.0116±0.0017	476904
WAG ≫ GCB	-0.0082±0.0015	476904
GCB ≫ LG	-0.0084±0.0017	476904

**Table 13:** Ranking and differences among MSAs

	***Δ***	***n***
non Metazoa		
Prank > Prograph	-0.0011±0.0006	1735825
Prograph ≫ Poa	-0.0289±0.0008	1735825
Poa ≫ Probabilistic	-0.0092±0.0007	1735825
Probabilistic > ClustalW	-0.0009±0.0007	1735825
ClustalW ≫ Mafft	-0.0028±0.0006	1735825
Mafft ≫ ClustalO	-0.0021±0.0005	1735825
ClustalO ≫ Probcons	-0.0043±0.0006	1735825
Probcons ≫ PartialOrder	-0.0107±0.0007	1735825
Metazoa		
Prograph ≫ Prank	-0.0451±0.0024	774969
Prank ≫ PartialOrder	-0.0382±0.0021	774969
PartialOrder ≫ Probabilistic	-0.0823±0.0023	774969
Probabilistic ≫ Mafft	-0.0210±0.0020	774969
Mafft ≫ Probcons	-0.0388±0.0015	774969
Probcons > ClustalO	-0.0032±0.0017	774969
ClustalO ≫ Poa	-0.0684±0.0020	774969
Poa ≫ ClustalW	-0.0885±0.0024	774969

**Table 14:** Output of the comparison of Mafft against Probcons over Metazoa

				
Metazoa:	Mafft_CodonDist_BioNJ - Probcons_CodonDist_BioNJ, 59613 OGs					
Metazoa:	Mafft_CodonDist_FastME - Probcons_CodonDist_FastME, 59613 OGs					
Metazoa:	Mafft_CodonDist_LST - Probcons_CodonDist_LST, 59613 OGs					
Metazoa:	Mafft_Gap - Probcons_Gap, 59613 OGs					
Metazoa:	Mafft_InducDist_BioNJ - Probcons_InducDist_BioNJ, 59613 OGs					
Metazoa:	Mafft_InducDist_FastME - Probcons_InducDist_FastME, 59613 OGs					
Metazoa:	Mafft_InducDist_LST - Probcons_InducDist_LST, 59613 OGs					
Metazoa:	Mafft_Parsimony - Probcons_Parsimony, 59613 OGs					
Metazoa:	Mafft_PhyML - Probcons_PhyML, 59613 OGs					
Metazoa:	Mafft_RAxML - Probcons_RAxML, 59613 OGs					
Metazoa:	Mafft_RAxMLG - Probcons_RAxMLG, 59613 OGs					
	Mafft is strongly better than Probcons					
	Mafft - Probcons: -0.0390 +- 0.0017, n=655743					

Sample output showing the selection of which methods to compare when summarizing results, Table 14. The difference of the Taxon measures is taken over corresponding pairs of trees. These corresponding pairs differ only in the components we want to compare. Furthermore, they will be computed over exactly the same population of OGs.

PhyML is the best tree builder using MSAs (Table 8). The results are consistent accross classes except for a significant worsening of Parsimony and Gap for Metazoa.

Global alignments [22] dominate the pairwise alignments methods (Table 9). The most significant difference between Metazoa and non-Metazoa is that CodonPAM is propelled to the front by a significant margin in Metazoa. It should be noted that the CodonPAM mutation matrix is an empirical mutation matrix based on data from vertebrates [23]. The genomes included in Metazoa have diverged more recently than for other classes, like Archaea, which also explains the better performance of the codon-based methods. From an information-theoretic point of view, codons are over an alphabet of size 61 as opposed to 20 for amino acids, so they must carry more information. Regardless of the reason, the advantage of codon-based methods is an order of magnitude larger than the differences between the other methods. Hence codon-based methods appear unavoidable for Metazoa. Table 10 confirms the same difference at the level of MSA-induced alignments.

The distance methods, Table 11, see LST changing from last position in non-Metazoa to first position in Metazoa. In this case the absolute differences are relatively small.

Table 12 shows the comparisons of empirical substitution matrices. The differences between the best and the worst matrix are statistically significant but very minor.

Table 13 shows the results for MSAs. PartialOrder, which is an algorithm designed to deal with alternative splicings, works better for Metazoa. ClustalW, from a middle ranking in non-Metazoa, drops to a clear last for Metazoa. The rest of the rankings remain quite consistent for all species.

The most important message coming out of these results is that the best methods are minimal evolution (distance) methods over pairwise alignments induced by MSAs. A method like Mafft_InducDist_BioME is 2-3 orders of magnitude faster than ML methods and outperforms them all by a good margin.

## Discussion

It may appear surprising that the best method for non-Metazoa starts by using Mafft which is not the best MSA (Table 13). In general, the best PTMS may not include the best components, and vice-versa, the best individual components may not give the best PTMS. Components may combine/exploit their abilities/weaknesses. For example, an MSA method which does a very good job with amino acids but a mediocre job with gaps, may compose very well with ML methods but poorly with Gap trees. We have to remember that the analysis of components, Tables 8, 9, 10, 11, 12 and 13 is done over an average of many situations.

The statistical significance of the difference between methods is one aspect, the magnitude of the difference is also important. The testing was done over such large samples that often minor differences are still statistically significant. We consider that a difference of less than *Δ*=0.01 (that is in 100 trees, on the average, we get one less error) is without practical significance. A *Δ*=0.05 difference, on the other hand, means that one method will produce one better branch every 20 trees, which can be considered significant.

Mafft_InducDist_BioME is the top method for non-Metazoa under the Taxon measure and is ahead of the top ML method, Prank_PhyML, by *Δ*=0.048 correct branches per tree. The number of incorrect branches per tree of each method is 1.425 and 1.473 respectively. (For Metazoa the best methods are PartialOrder_CodonDist_BioNJ and Prank_RAxMLG with errors 2.281 and 2.555 respectively.) This shows that there is a long way to perfection. Some of this distance (the 1.425 or 2.281 in these cases) is due to the inherent randomness of the molecular mutations left by evolution, some of it may be due to imperfect PTMSs.

### Caveats, what can go wrong?

Here we describe some problems that may affect the power/correctness of the PTMS evaluation (for dependencies on absolute/relative distances, number of leaves and sequence length see Methods). 

· The OGs should follow the same evolutionary history. This is normally the case, except when we have lateral gene transfers (LGT) or OGs which do not follow Fitch’s definition of orthology [24] precisely. For the purpose of testing the methods, it is much better to skip dubious OGs. The OMA orthologous database fits best our needs [25,26], as it sacrifices recall in favor of precision.

· The Intra test measures the ability of recovering a phylogenetic signal from sequences. Other reasons for mutation of the sequences may leave their trace in the conclusions. For example, it is known that the environmental temperature affects the GC content of the sequences due to DNA stability [27]. Consequently, we will expect a bias at the codon level that will tend to group together organisms that live in a high-temperature environment.

· The methods should produce trees with complete structure, i.e. no multifurcations, all nodes must be binary. A method which produces a tree with multifurcations will have an advantage as it will normally make fewer mistakes. In the extreme, a star tree is always correct.

· Since PTMSs have been in use for many years, the preferred methods of the community may show an undeserved good performance under the Taxon measure (but not under the Intra measure!). This is not unreasonable, since for bacteria many classical phylogenetic methods do not apply (e.g. bacterial paleontology has very few results), and taxonomies may have been constructed with some of these methods. A method which has been the favourite of the taxonomists will be displaced to the left of the main line in Figure 3 (it reduces the Taxon measure and leaves the Intra unchanged). We can see that parsimony, RaxML and PhyML show a small shift to the left and hence it is possible that these methods have biased the building of the Taxonomies. This shift is noticeable but quite small, so we can conclude that this is not a major bias.

**Figure 4:**
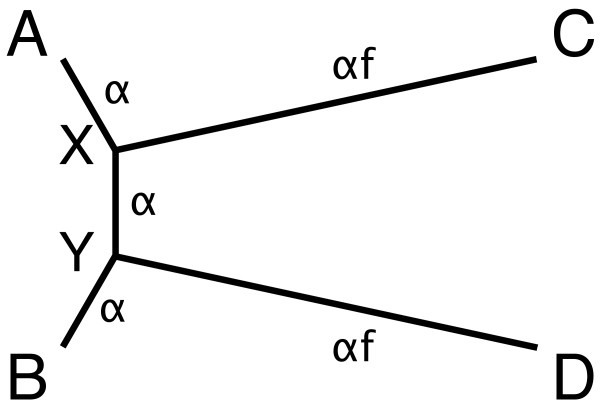
An extreme tree with 4 leaves illustrating the problems of Long Branch Attraction (LBA).

· Finally, the Intra measure, by being a consistency measure, may be insensitive to systematic mistakes of the tree building. This would be something that affects the Intra measure but not the Taxon measure. Stephane Guindon suggested that long branch attraction (LBA) could mislead the Intra measure for methods that suffer from it, by systematically computing one of the incorrect trees. To study this properly we generated a new class called LBAExamples which is composed of a quartet ((A,C),(B,D)), where the branches leading to the leaves C and D are much longer than the other branches. This quartet is sometimes called the “Felsenstein example” and is used to demonstrate how some methods, like parsimony, systematically will reconstruct the wrong tree (and hence the name LBA). See Figure 4.We built 500 such quartets for random values of *α*(the length of the shortest branches) and for 5 different sets of leaves C and D with the values *f*=5,7,5,10,12.5 and 15 (the ratio of the long branches to the short branches). That is 100 examples of each quartet. The results are available in their entirety in the same website repository as all the other results. The most significant results of the study of these quartets are:

· 

There is a clear division of methods under the Taxon measure (in this case the taxonomic tree is the correct topology) and the Intra measure. All the methods using Parsimony, Gap, SynPAM and PrankGuide suffer from LBA (and score a high value, Taxon ≥ 0.16 and Intra ≥ 0.252). All the other methods (which do not suffer from LBA) score much lower, Taxon ≤ 0.036 and Intra ≤ 0.0697. Remember that there are only 3 possible quartets and that an error in a quartet gives a distance of 1, hence for quartets the values of the Taxon measure coincide with the number of incorrect trees. The gap that separates the non-LBA from LBA methods is large in absolute and in relative values. So we can conclude that LBA is successfully detected by both measures.

Methods based on k-mer statistics (not reported here, but also evaluated in our computations) fare much worse than all the other methods in general. These are methods which count the number of, for example, tri-mers, and use as distances a statistical test (like chi-squared) on the tri-mer frequencies. For example, a method based on DNA tetra-mers scores 0.952 for the Taxon measure (it gets 95.2% of the quartets wrong!) but scores 0.0917 in the Intra measure. This is quite extreme, (the method is very poor in every context) but supports the observation that the Intra measure is a consistency measure and if the method systematically fails and there is only one way of failing, then the consistency is good. In terms of the plot of Figure 3, these cases will be points displaced to the right of the main line. This extreme case reinforces our recommendation of using both measures to conclude the performance of methods.

This side study showed additional surprises. The guide tree produced by Prank, usually quite good, but unfortunately it suffers severely from LBA (Taxon = 0.532). We were also unaware that the SynPAM methods, which are maximum likelihood methods, also suffer from LBA.

The above caveats indicate that the problems are relatively few and seldom apply to both measures. Consequently a method which does well under both measures is a very strong candidate.

## Conclusions

We show, through a comparison of methods against trees involving tens of millions of data points, which are the most effective PTMSs. This uncovers a big surprise as one of the favorite methods among the community, the ML methods, score poorly. Methods based on MSA induced pairwise alignments and minimal evolution not only produce better trees, but are 100 to 1000 times faster to compute. This should revolutionize this niche of bioinformatics.

We also show that a new measure of quality, the Intra measure, is highly correlated with the Taxon measure (closeness to taxonomic trees) and it does not suffer from the biases of the practice. These new measures are likely to be extremely helpful in the development of new and better algorithms.

## Methods

We cannot show all the computations and results in this publication because of their size (about 57Gb). We have developed a web site which allows the exploration of all the data and all the results to their most minute detail. We intend to maintain this website for as long as possible, and to upgrade it periodically both with new genomes and with new methods. It contains very useful information in our view. This can be found in: 

### Source data

The study was done over complete genomes for three reasons: species coverage is quite ample, 755 complete genomes were used, we can obtain a large number of very reliable OGs and since all OGs from entire genomes are used, no selection bias is possible. A complete description of the classes can be found in the OMA1000 database which is accessible at: 

To do the analysis, the genomes were grouped in the classes shown in Table 6 (in this work we will call any of these groups a “class”). The column “OGs kept” shows the number of groups which had 4 or more acceptable sequences.

The study includes all the publicly available genomes as of Nov/2010, the release of OMA1000 [21]. For proteobacteria and firmicutes, which are relatively overrepresented and many species sequenced multiple times (e.g., there are 26 genomes of different strains of E.coli), only 265 genomes of 452 were chosen for proteobacteria (89 of 177 for firmicutes) as follows: For each pair of genomes an average evolutionary distance was computed. Iteratively, one of the members of the closest pair of genomes was discarded. The discarded one was the one with lower “quality index” (a simple ad-hoc measure of quality of complete genomes). In this way we retained the “best”, most diverse, 265 proteobacteria and the most diverse 89 firmicutes. All the major versions of model organisms ended up in these classes. As a control, we also computed the same trees over all the genomes of firmicutes (177). The correlation coefficient of the Taxon measure between the full class of all firmicutes and the class with 89 genomes is 0.994058. Knowing this value we are confident that the results are not affected by removing very similar (or repeated) genomes.

Comparing within classes is better than grouping the classes together for the following reasons: 

· The classes are more uniform and may reveal biases (as they do) specific to the classes.

· The missing relations - between classes - are usually so obvious that almost no method will get them wrong. It is the fine grain differences that matter.

· The computation time would be out of reach for some methods.

· The problems of some well documented LGTs, like proteins of mitochondrial origin, are avoided.

The selection of the OMA[20] database of OGs was done because OMA is particularly careful about removing paralogous sequences at the expense of sometimes splitting groups (precision at the expense of recall). A split OG is a minor loss of data, of little consequence given our sample size, whereas the inclusion of paralogous sequences breaks the basic assumption for the correctness of the Taxon and Intra measures. The main assumption that links the Intra measure with the quality of the methods is that any pair of groups represents the same evolution path. If one group contains an orthologous pair and the corresponding pair in another group is paralogous, these will correspond to different evolutionary histories and the comparison is wrong.

### Sequence/group cleanup

Only the OGs with 4 or more sequences are used (2 or 3 sequences will never show different unrooted topologies). We also removed all but one copy of identical sequences. A method which is given a few identical sequences will most likely place them all in a single subtree. The shape of this subtree will be unrelated to the phylogeny of the sequences (there is no information available to make a decision). Since this adds noise to the results (not necessarily bias), we remove all identical sequences but one from the OGs. Additionally we remove sequences for which more than 5% of their amino acids or codons are unknown (“X”) as this is a sign of poor quality of the sequence. Both policies together remove about 3.5% of the sequences.

### Bayesian methods

Bayesian methods for tree building have not been included in this study because they do not follow the PTMS definition. In principle a bayesian tree building method produces a probability distribution over all trees given the corresponding priors. If the priors are ignored and only the tree with highest probability is selected, then this is ML, not bayesian. Approaches which build consensus trees from several of the most probable trees produce multifurcating trees which contain less information and hence are not comparable to fully determined trees. Any prior which contains information about the tree which is not extracted from the sequences themselves will violate our assumptions for PTMS.

### Tree building methods

We have computed 176 trees per OG, that is a total of 176 × 193138 trees or about 34 million trees. The tree building methods are a combination of several components, for example Mafft_PhyML represents the method composed of building an MSA with Mafft and then using the PhyML program. The component methods are only a subset of the existing methods. The ones chosen are the ones that we perceive as the most popular and effective in the community plus the ones which have been written locally. We welcome suggestions of promising new components to test.

Multiple sequence alignment methods from amino acid sequences: 

· ClustalW - a widely used MSA program based on a guide tree computed from pairwise alignments, version 2.0.10 [28]

· ClustalO - a recent improvement of ClustalW, [29]

· Mafft - a rapid MSA based on fast Fourier transforms, version 6.843 [30,31]

· PartialOrder - A method based on partial order graphs, currently being developed at the CBRG in the ETH Zurich, designed to accommodate alternative splicings.

· Poa - a progressive multiple sequence alignment based on a graph representation where each new sequence is aligned by pairwise dynamic programming [32]

· Prank - a phylogeny-aware gap placement MSA, version 100802 [33]. The guide tree used by Prank has its own merits and is used as one of the possible trees, under the name PrankGuide.

· Probabilistic - A method based on probabilistic ancestral sequences developed for the Darwin system. [34-36]

· Probcons - a probabilistic consistency-based MSA, version 1.12 [37]

· Prograph - a method of progressive graph alignment, similar to Prank, currently under development at the CBRG in the ETH Zurich.

Methods which produce a tree from an MSA: 

· Gap - produce a tree by parsimony, replacing all amino acids by a single symbol [19]. In this way the only information left is gap or no-gap.

· Parsimony - equal character cost, counting gaps as a special character, as implemented in Darwin [36]

· PhyML - a fast and accurate heuristic for estimating ML phylogenies, version 3.0 [38,39], used with gamma corrections and the LG [40] matrices.

· RAxML - randomized accelerated ML for high performance computing, version 7.0.4 [41], uses a substitution matrix described in [42]

· RAxMLG - randomized accelerated maximum likelihood for high performance computing, version 7.0.4 [41], used with gamma corrections and a substitution matrix described in [42]

Pairwise alignment methods which compute a distance and variance matrix from amino acid or coding-DNA sequences. Every sequence is aligned to every other sequence. In all cases, after the alignments are done, the distances between pairs of sequences are estimated by ML: 

· GlobalCodonPAM, LocalCodonPAM - global/local alignments using a codon substitution matrix (61×61) [23]

· GlobalJTT, GlobalLG, GlobalWAG, GlobalGCB, global alignments [22] using the JTT [43], GCB [44], WAG [45] and LG [40] substitution matrices

· LocalJTT, LocalLG, LocalWAG, LocalGCB, local alignments [46] using the JTT [43], GCB [44], WAG [45] and LG [40] substitution matrices

· LogDelGlobal, LogDelLocal - global/local alignments using GCB and a special deletion cost function based on the observed zipfian distribution of gap lengths [47].

· GlobalSynPAM, LocalSynPAM - global/local alignments using a codon substitution matrix (61×61) which ignores all mutations except the synonymous ones [48].

Pairwise alignment methods which compute a distance and variance matrix from the sequences in an MSA: 

· CodonDist - estimate the ML CodonPAM distance from pairwise alignments induced by an MSA. The MSA is over amino acids, and the corresponding codons from the protein are used to replace the amino acids. [23]

· InducDist - estimate the ML distance from pairwise alignments induced by an MSA with the GCB rate matrices.

Distance methods which produce a tree from a distance/variance matrix: 

· BioNJ - an improved version of the NJ algorithm, [49].

· FastME - build a tree using the minimum evolution principle, version 2.07, [50].

· BioME - a version of FastME with iterative improvements

· LST - build a tree using the least squares principle, with the distances weighted by the inverse of their variance [36,51].

· NJ - the neighbor joining method, [52].

### Taxonomies database

We chose the NCBI [53,54] taxonomies to build the taxonomic trees, the basis of our Taxon measure. The NCBI database is detailed and extensive and it covers all the species that were included in OMA1000. The ITIS database[55], another well known taxonomic database, is not as complete, in particular for bacteria, where many of the entries we need are absent.

### Computation

The computations were carried out in our own cluster of Linux machines, about 300 cores. These were done using Darwin [36] as a host system. Additionally we used the Brutus cluster, a central service of ETH. We estimate that we used about 646,000 hours of individual CPUs. Table 15 shows the top most time consuming tasks.

**Table 15:** Top uses of cpu time

**Task**	**cpu**
PhyML	135833 hrs
RAxMLG	111432 hrs
Parsimony	86882 hrs
Intra measure	63713 hrs
Prank	54101 hrs
RAxML	34673 hrs
PartialOrder	20132 hrs
Gap	8707 hrs
Taxon measure	5389 hrs

Of the classes, most of them took time proportional to the number of OGs. The exception being Metazoa, which has bigger OGs and longer sequences. As a consequence the lion’s share of computation was taken by Metazoa.

### Correlations as the main test

As mentioned above, the Intra and Taxon measures are not directly comparable (they are expected values over very different populations). Any of the measures is not comparable accross classes of species either. This is shown to be the case with Metazoa which behaves differently to the others classes. The distances are also radically different for different classes. On the other hand we are always measuring an average RF distance, hence there should be a linear relation between the different measures for different classes when comparing the different PTMSs. In other words, a suitable comparison could be through a linear transformation or a linear regression of one into the other. For the regression, the coefficients are not important, the quality of the fit is the important aspect. This is exactly what is captured by Pearson’s correlation coefficient, and hence this is the main tool we use to compare measures of different PTMS accross different populations.

### Distances between trees

We use the Robinson-Foulds (RF) [17] distance to measure distances between trees. The RF distance basically counts how many internal branches of the unrooted trees do not have a corresponding branch which divides the leaves in the same two sets. For trees with *n* leaves, the RF distance may be as high as *n*−3.

When the taxonomic tree is not completely determined (that is, some nodes have more than two descendants), we have to correct the computation of the RF distance. This is relatively straightforward to fix. The maximum distance in these cases is less than *n*−3.

### Absolute vs relative distances vs 0-1 distances

There are arguments to use the absolute RF distance and arguments to use relative RF distances (the absolute distance divided by *n*−3). Fortunately, the results are remarkably consistent for the absolute and relative RF distance. Table 16 shows Pearson’s and Spearman’s correlation coefficients between the absolute and relative measures, per class for all PTMSs. Clearly the rankings are not affected by this choice.

**Table 16:** Correlations between the absolute and relative distances (all leaves)

	**Taxon**		**Intra**	
**Class**	**Pearson’s**	**Spearman’s**	**Pearson’s**	**Spearman’s**
Actinobacteria	0.9963	0.9171	0.9988	0.9888
Archaea	0.9969	0.8825	0.9978	0.9414
Firmicutes	0.9973	0.9765	0.9997	0.9973
Metazoa	0.9851	0.9775	0.9893	0.9574
OtherBacteria	0.9827	0.9570	0.9965	0.9736
OtherEukaryota	0.9944	0.8710	0.9948	0.9490
Proteobacteria	0.9946	0.8834	0.9993	0.9852

There are also arguments that the RF distance may not reflect evolutionary distance. That is to say, that sometimes a small evolutionary change produces a tree which has a large RF distance to the original and other times a large evolutionary change produces a tree which has a small RF distance. This has been recently discussed in [56]. To take this concern in consideration we also computed the 0-1 distances, a Hamming-style distance, 0 if the trees are equal, 1 otherwise. The 0-1 distance may not be as sensitive as other distances, since it collapses all wrong trees into a single case, in particular it will give very little information about large trees when there is almost always some error. The correlation coefficients between the 0-1 distance and the RF distance for the Taxon measure are 0.9905 (non Metazoa) and 0.9004 (Metazoa). The correlation is excellent for non Metazoa, and the rankings for the Taxon or 0-1 distances have relatively insignificant differences. The correlation for Metazoa is good but lower and some methods, notably ML methods, move ahead. If we take as an example in Metazoa, we find that its 0-1 distance is 0.5417. Excluding the trees with 10 or less leaves, it is 0.9614 and excluding the trees with 20 or less leaves it is 0.9824. Even medium size trees are mostly wrong. Clearly the 0-1 measure loses too much information for large trees and reflects the quality of small trees alone. This has motivated us to study the impact of the distance functions used for the Taxon and Intra measures in depth, which will be reported in a future work. The 0-1 distances are shown as a separate column in the full Tables 1 and 14. To make safer conclusions about comparisons of methods, we should use the Taxon, Intra and 0-1 measures.

### Large trees vs small trees

It may be argued that small trees are too simple and bigger trees are the important ones. To analyze this effect we divide the OGs into two groups, the ones with 15 or fewer leaves and the ones with more than 15 leaves. We then compute the correlation coefficient for the measures on all PTMS for these two groups. Table 17 shows the results for each of the classes. All the correlations are high, and those for the non Metazoa are remarkably high. From these correlations we can conclude that the number of leaves used for the quality analysis does not influence the results.

**Table 17:** Correlation of the Taxon measure of all PTMS for OGs with 15 or less leaves and the rest

**Class**	**Pearson’s**
Actinobacteria	0.9930
Archaea	0.9925
Firmicutes	0.9933
Metazoa	0.9143
OtherBacteria	0.9785
OtherEukaryota	0.9846
Proteobacteria	0.9887

### Long sequences vs short sequences

In a similar way it may be argued that groups with long sequences behave differently than groups with short sequences. To analyze this effect we again divide the OGs into two groups, the ones with average sequence length less or equal to the median and those with average above the median. We then compute the correlation coefficient for the measures on all PTMS for these two groups. Table 18 shows the results for each of the classes. As above, the correlations are high and even higher for non Metazoa. From these correlations we can conclude that the average length of the sequences used for the quality analysis does not influence the results. In these last two comparisons, where we select the groups based on properties of the OG (like number of sequences and average length), we have to use the Taxon measure which is based on distances of a single group. The Intra measure is based on pairs of groups, and hence not suitable for these splits.

**Table 18:** Correlation of the Taxon measure of all PTMS for OG above and below the average sequence length median

**Class**	**median**	**Pearson’s**
Actinobacteria	305.4	0.9971
Archaea	258.0	0.9902
Firmicutes	283.2	0.9950
Metazoa	360.0	0.9600
OtherBacteria	309.1	0.9907
OtherEukaryota	419.3	0.9928
Proteobacteria	295.6	0.9972

### Variance reduction techniques

To compare two building methods in the Taxon measure, we can use the average distances to the taxonomic tree over all the OGs. These averages will have a relatively large variance and the difference may not be statistically significant. To refine the comparison of two particular methods, we study the difference of distances of the two methods for each OG. The expected value of the difference coincides with the difference of the averages, but the confidence margins are much better because the variance of the difference is normally smaller. This is a well known variance reduction technique [57].

## Competing interests

The author declares that he has not competing interests.
